# Proteome Profiling of PMJ2-R and Primary Peritoneal Macrophages

**DOI:** 10.3390/ijms22126323

**Published:** 2021-06-12

**Authors:** Alexander L. Rusanov, Peter M. Kozhin, Olga V. Tikhonova, Victor G. Zgoda, Dmitry S. Loginov, Adéla Chlastáková, Martin Selinger, Jan Sterba, Libor Grubhoffer, Nataliya G. Luzgina

**Affiliations:** 1 V. N. Orekhovich Research Institute of Biomedical Chemistry, Pogodinskaja Str. 10, 119121 Moscow, Russia; kozhinpm@gmail.com (P.M.K.); tiolika@gmail.com (O.V.T.); victor.zgoda@gmail.com (V.G.Z.); dmitry.loginov@biomed.cas.cz (D.S.L.); ngluzgina@gmail.com (N.G.L.); 2 Faculty of Science, University of South Bohemia, Branišovská 1760, 370 05 České Budějovice, Czech Republic; adelachlastakova@seznam.cz (A.C.); selinm01@prf.jcu.cz (M.S.); sterbaj@prf.jcu.cz (J.S.); liborex@paru.cas.cz (L.G.); 3 BioCeV—Institute of Microbiology of the CAS, Prumyslova 595, 252 50 Vestec, Czech Republic; 4 Institute of Parasitology, Biology Centre of the Czech Academy of Sciences, Branišovská 31, 370 05 České Budějovice, Czech Republic

**Keywords:** PMJ2-R, peritoneal macrophages, phagocytosis, proteome, LC-MS/MS

## Abstract

In vitro models are often used for studying macrophage functions, including the process of phagocytosis. The application of primary macrophages has limitations associated with the individual characteristics of animals, which can lead to insufficient standardization and higher variability of the obtained results. Immortalized cell lines do not have these disadvantages, but their responses to various signals can differ from those of the living organism. In the present study, a comparative proteomic analysis of immortalized PMJ2-R cell line and primary peritoneal macrophages isolated from C57BL/6 mice was performed. A total of 4005 proteins were identified, of which 797 were quantified. Obtained results indicate significant differences in the abundances of many proteins, including essential proteins associated with the process of phagocytosis, such as Elmo1, Gsn, Hspa8, Itgb1, Ncf2, Rac2, Rack1, Sirpa, Sod1, C3, and Msr1. These findings indicate that outcomes of studies utilizing PMJ2-R cells as a model of peritoneal macrophages should be carefully validated. All MS data are deposited in ProteomeXchange with the identifier PXD022133.

## 1. Introduction

Macrophages and macrophage-like cells are common objects of research in the physiology of immune cells and phagocytosis in health and disease. For these purposes, both primary and immortalized cell lines can be used.

Primary cells accurately model our cell type of interest’s behavior, as their structural and functional characteristics in vitro almost completely match those of the cells in vivo [1]. However, these cells can divide only a limited number of times, and the phenotype of the cells from different passages can be altered, reflecting their functional state [1]. Additionally, the phenotype of primary cells depends on the origin of the cells (isolated from different laboratory animals or even from different organs of the same animal, those obtained from biological samples of different patients, etc.) due to the individual characteristics of organisms [2]. These factors determine the heterogeneity/non-standardization of such research objects.

This disadvantage is lacking in immortalized cell lines. Such cells divide an unlimited number of times, which determines their high standardization [2]. However, their phenotype differs from the primary cells due to genotype changes. Thus, a phenotype comparison of immortalized and normal cells is necessary before using the former as models.

Various immortalized macrophage-like mouse cell lines are widely used in biomedical research, including RAW 264.7 (ATCC^®^ TIB-71™), Wehi-3 (ATCC^®^ TIB-68™), J774A.1 (ATCC^®^ TIB-67™), IC-21 (ATCC® TIB-186™), etc. [3,4,5,6,7,8,9]. For example, the macrophage-like cell line RAW 264.7, obtained from Balb/c mice injected with the Abelson murine leukemia virus, has been widely used in the research of phagocytosis and macrophage physiology [5]. In recent years, its proteomic analyses have been performed, including studying a large-scale proteome, phagosomal proteomics [6,7], and responses to cytokines [8,9]. Guo et al. [5] has performed an in-depth comparative proteomic study of phagosomes isolated from RAW 264.7 and normal mouse bone marrow-derived macrophages, with a quantitative analysis of more than 2500 proteins. Authors have described significant differences in abundances of a large number of proteins, including the most important cellular receptors involved in phagocytosis, and suggested a careful use of the RAW 264.7 cell line when studying the functions of phagosomes and the process of phagocytosis [5]. Thus, comprehensive studies of the physiology of such cells, including proteomics, are necessary for the careful characterization of their phenotypes.

Only a few immortalized macrophage-like mouse cell lines have been derived from peritoneal macrophages (PM). One of them is PMJ2-R (ATCC CRL2458)—an immortalized PM line obtained from C57BL/6 mice by in vivo infection with retrovirus J2 carrying the v-raf and v-myc oncogenes [10]. These immortalized cells have many morphofunctional characteristics of PM, such as the characteristic of cell surface antigens, LPS-induced secretion of IL6, but not IL1 and TNF-a. The PMJ2-R cells are highly phagocytic, contain nonspecific esterase, and express the Fc receptor, F4/80, Mac-1, Mac-2, and Ly5.1. High expression levels of MHC class II antigens can be induced by recombinant murine interferon-gamma. Thus, cells exhibit many, but not all, functions and features of primary macrophages [10]. It was also demonstrated that PMJ2-R cells can be used for the investigation of an initial development phase of tick-borne encephalitis in macrophages [11].

PMJ2-R was derived from the PM of the C57BL/6 mice, unlike many other immortalized cell lines, including RAW 264.7, Wehi-3, and J774A.1. These mice have been described to have a different immune response compared to others, particularly to BALB/c and CBA mice [12]. C57BL/6 mice are characterized by a higher number of myelokaryocytes compared to CBA mice. Furthermore, their peripheral blood contains a higher number of leukocytes, monocytes, and lymphocytes, and a higher content of Kupffer cells in the liver has been observed in these animals. This indicates a predominance of cellular elements (monocytes, macrophages, Kupffer cells) in the mononuclear phagocyte system (bone marrow-peripheral blood-liver) of C57BL/6 mice. Subsequently, this reflects a genetically determined higher power (quantitatively) of the system of mononuclear phagocytes in C57BL/6 mice compared to CBA mice [13]. It is believed that classical type activation (M1) is more characteristic in macrophages of C57BL/6 mice in contrast to the alternative one in macrophages of BALB/c and CBA mice [14,15,16].

This study aims to compare the proteomes of PMJ2-R cells and primary peritoneal macrophages isolated from C57BL/6 mice, including the analysis of proteins associated with phagocytosis.

## 2. Results

High-throughput shotgun proteomic analysis of both cell types was performed to compare PMJ2-R and PM proteomes. A total of 4606 proteins were identified, 4005 of which were identified by two or more peptides. A total of 796 proteins were quantified by Progenesis software (see Supplementary File 1), out of which 441 were significantly changed in PMJ2-R cells, including 49 and 392 proteins that were up- and downregulated, respectively.

Table 1 shows the top 15 proteins with either increased (more than 60 times) or decreased (more than 70 times) abundances in immortalized cells. Many proteins listed in this table are involved in processes associated with nucleic acid binding and processing, as well as cytoskeleton organization and metabolism.

**Figure 1:**
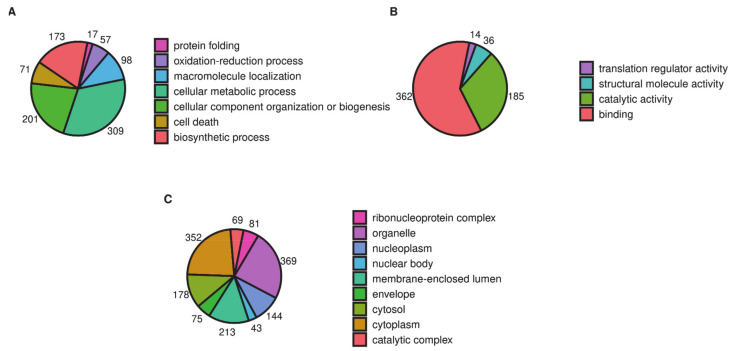
Functional classification of up- and downregulated proteins in PMJ2-R cells according to the Gene Ontology database: Biological process (**A**), molecular function (**B**) and cellular localization (**C**). Pie charts were built using significantly enriched GO terms.

Gene Ontology classification of all changed proteins also demonstrates participation in binding processes (362 proteins, including 136 proteins responsible for nucleic acid binding), cellular metabolic processes (309 proteins), and cellular component organization or biogenesis (201 proteins). Figure 1 shows schemes of Gene Ontology classification for proteins with changed abundance in PMJ2-R cells in terms of biological process (Figure 1A), molecular function (Figure 1B), and cellular localization (Figure 1C).

**Figure 2:**
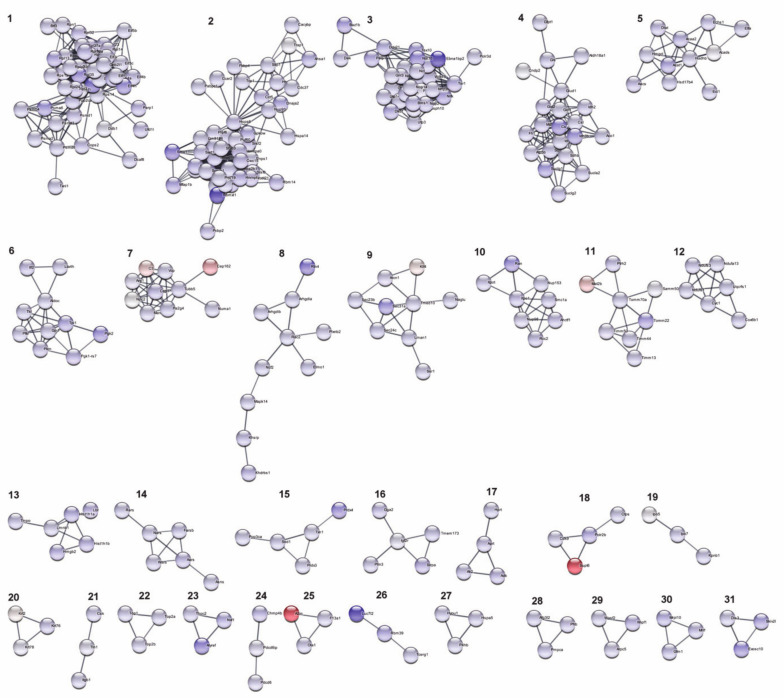
STRING network of PMJ2-R proteins with significant regulation levels. Expression ratios were mapped to the nodes using blue-red gradient, where blue is related to the downregulated and red to the upregulated proteins. Proteins without interaction partners were omitted from the visualization. Network edges represent the confidence of interaction. The required interaction score was set to >0.7.

Analysis of PMJ2-R differently expressed proteins using STRING resulted in clusters based on protein interactions (Figure 2). The largest clusters were formed by downregulated proteins involved in protein synthesis processes, including translation (Figure 2, cluster 1), splicing, and processing of mRNA (Figure 2, cluster 2), biogenesis of ribosomes, and metabolic processes of RNA (Figure 2, cluster 3). Several clusters were formed by proteins associated with cell metabolic processes, such as tricarboxylic acid cycle and glycolysis (Figure 2, clusters 4,6), fatty acid metabolism (Figure 2, cluster 5), and NADH dehydrogenase activity (Figure 2, cluster 12).

Surprisingly, highly upregulated proteins, namely Supl6 and A2m (corresponding fold changes of 190 and 179, respectively), were assigned to the clusters with downregulated proteins (Figure 2, clusters 18 and 25, respectively). It was shown that Supt6 (the most crucial factor of transcription elongation and RNA processing) interacts with proteins Cdk2 and Polr2b, which are related to positive transcription elongation factors, and A2m (a proteinase inhibitor) interacts with the proteins Ola1 (OBG-like GTPases) and F13a1 (subunit A coagulation factor XIII).

Among detected proteins, 19 belong to the process of phagocytosis, of which seven proteins (Anxa3, Arhgap25, Cd47, Cdc42, Lman2, Nckap1l, Pld4) were produced by immortalized cells at the comparable level with the primary ones. The abundance of 10 proteins (Csk, Elmo1, Gsn, Hspa8, Itgb1, Ncf2, Rac2, Rack1, Sirpa, Sod1) in PMJ2-R cells was decreased, while the content of C3 and Msr1 was increased.

**Figure 3:**
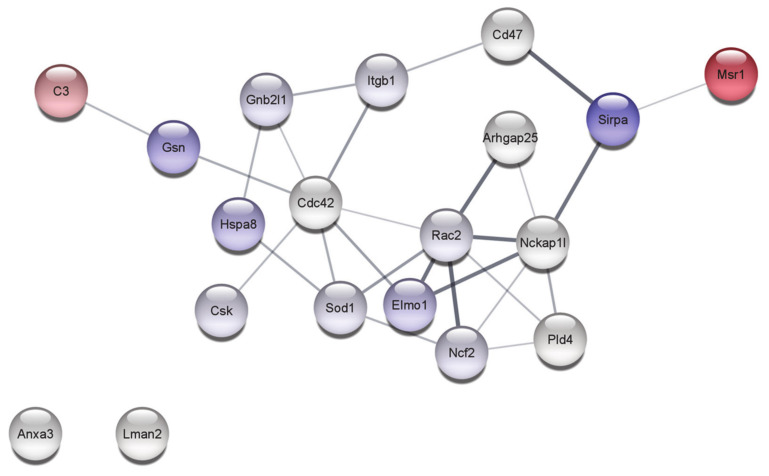
STRING network of PMJ2-R proteins involved in phagocytosis. Expression ratios were mapped to the nodes using blue-red gradient, where blue is related to the downregulated and red to the upregulated proteins. Network edges represent the confidence of interaction. The required interaction score was set to >0.4.

An interaction network of identified proteins associated with the process of phagocytosis in PMJ2-R cells is shown in Figure 3.

**Figure 4:**
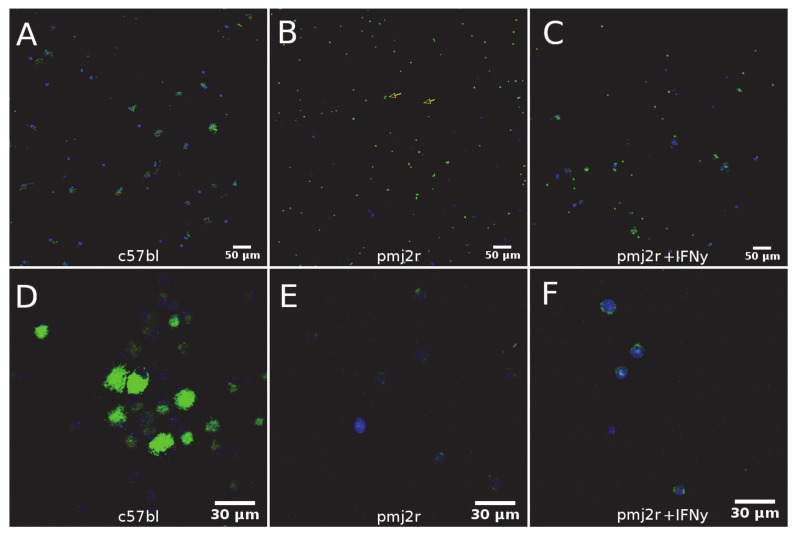
Functional activity of PMJ2-R and PM (C57BL/6) cells. Cells were incubated with fluorescently conjugated zymosan, and their ability to bind to the particles was examined by fluorescence microscopy: (**A**–**C**) Cells were incubated with FITC-dextran, and accumulation of the labeled particles was studied using fluorescence microscopy; (**D**–**F**) Green–FITC-labeled particles, blue-DAPI-stained cell nuclei.

To confirm the obtained proteomic data, a study of cell functional activity was performed. The ability of PMJ2-R cells to Fc-receptor or complement-receptor-mediated phagocytosis of opsonized zymosan particles [17] was dramatically reduced (Figure 4A–C). It should be noted that (based on FITC-dextran capture) the endocytic ability of PMJ2-R cells [18] was also decreased (Figure 4D–F, Table 2). Phagocytosis in IFN-gamma activated PMJ2-R cells was slightly increased (Figure 4B,D), but the rate of endocytosis remained unchanged (Figure 4E,F, Table 2).

## 3. Discussion

Macrophages are key components of the innate immune response. These cells possess a diverse array of receptors that enable responses to various external stimuli, including cytokines, chemokines, and molecules associated with pathogens [2,19,20]. Signals resulting from these stimuli activate a range of functional responses of macrophages, such as adhesion, migration, phagocytosis, proliferation, survival, cytokine release, and production of reactive oxygen and nitrogen species [19,20].

In this regard, phenotype comparison of PMJ2-R cells and PMs by comparing the proteins associated with innate immunity, particularly those involved in the process of phagocytosis, is of particular interest.

PMJ2-R cells were enriched in proteins C3 (61.62 times) and Msr1 (839.75 times) compared to normal PMs. C3 is a protein of the complement system, a cascade of proteolytic reactions required for the humoral defense of the body against foreign agents. It is an important component of the innate and adaptive immunity stimulating the phagocytosis of microbial cells, on the surface of which the complement system is activated. C3 proteolysis is a key event in this activation that is observed in all known activation mechanisms. Different expression levels of C3 protein have been shown for inactive and activated macrophages [21,22]. In particular, high abundances of C3a and C5a proteins are characteristic of alternatively activated macrophages (M2) [23].

At the same time, the expression of Gelsolin (Gsn) protein functionally linked to C3 in PMJ2-R cells turned out to be reduced (Figure 3). Gsn is a calcium-dependent actin severing and capping protein and is required for the efficient phagocytosis mediated by IgG but not the complement system [24]. There is a reason to believe that gelsolin is a part of the molecular mechanism that distinguishes between complement- and IgG-mediated phagocytosis. The latter requires a more dynamic restructuring of the cytoskeleton [24]. The abundance of this protein and HSP70-family protein Hspa8 catalyzing clathrin turnover was reduced in the PMJ2-R cells. During phagocytosis, phagocytic receptors and membrane material must be inserted into the phagosome membrane since it is located above the phagocytic target. This implies an active participation in the process of phagocytosis of the recirculation mechanism mediated by clathrin. Blockade of hsc70, which catalyzes clathrin turnover, leads to inhibition of IgG-opsonized objects phagocytosis [25].

Integrin β1 (Itgb1), whose abundance was decreased in PMJ2-R cells, also plays a vital role in the regulation of IgG3-mediated phagocytosis as an element of the SLO/β1 integrin/NOX2/ROS pathway. Blocking integrin β1 selectively prevents the binding of murine IgG3 (mIgG3) to macrophages. At the same time, manganese, an integrin activator, increases the binding activity of mIgG3 with macrophages. Thus, Itgb1 functions as a part of the IgG receptor complex [26,27].

Protein Msr1 is associated with the process of alternative activation of macrophages. It belongs to the class A macrophage scavenger receptors and has several isoforms resulting from alternative splicing. The defining property of scavenger receptors is the ability to bind and remove modified low-density lipoproteins. They are involved in a wide range of biological processes, including apoptosis and pathogen clearance. It has been shown that the functional activity of Msr1 can determine the ability of macrophages to polarize [28]. Thus, in macrophages activated by IL-4 (alternatively activated macrophages M2), polyubiquitylation of the MSR1 receptor K63 leads to the recruitment of the TAK1/MKK7/JNK signaling complex, thereby facilitating the phenotypic transition from an anti-inflammatory to a pro-inflammatory state, which is terminated after MSR1 deletion or JNK inhibition. Alternatively, activated M2 macrophages play an important role in maintaining tissue homeostasis by removing dead cells, cellular debris, and lipoprotein aggregates through phagocytosis. A proteomic study of the alternatively activated macrophages has shown enhanced production of proteins involved in homeostatic functions such as proteolysis, lipolysis, and transport of nutrients [28]. One may conclude that increased expression of C3 and MSR1 proteins indicate that PMJ2-R cells may be more prone to alternative polarization than normal mouse PM. This issue should be addressed in further studies.

The expression of a signaling regulatory protein alpha (Sirpa) protein which was functionally linked to Msr1 in PMJ2-R cells was reduced (Figure 3). This may indicate changes in the effector function activity of innate immunity and the ability to phagocytose host cells caused by this protein. Sirpa acts as an inhibitory receptor and interacts with the transmembrane protein CD47, also called the “do not eat me” signal for phagocytic cells. In this case, the negative regulation of phagocytosis is carried out due to Sirpa accumulation on the macrophage membrane in the phagocytic synapse region, where Sirpa–CD47 interaction occurs. Cancer cells express CD47 in significant amounts, resulting in Sirpa activation and inhibition of macrophage-mediated destruction. In this regard, developed anticancer therapeutic strategies aim to inhibit CD47 and/or SIRPα [29]. The abundance of CD47, which is expressed by all healthy cells of the host [30], was comparable between PMJ2-R and PM.

In PMJ2-R cells, the expression of proteins Ncf2 and Sod1 associated with the production of reactive oxygen species was decreased. The N*cf2* gene encodes p67phox, an essential component of the multiprotein enzyme NADPH oxidase in phagocytic cells responsible for the production of reactive oxygen species during phagocytosis [31].

Phagocytes undergo noticeable changes in the way they process oxygen under certain stimuli [32]. An increase in the oxygen uptake rate is accompanied by the production of large amounts of superoxide and hydrogen peroxide. Thus, oxygen is used to produce potent microbicidal agents after the initial superoxide formation [32]. At the same time, glucose is oxidized through the hexose-monophosphate pathway to re-generate NADPH consumed during the reduction in molecular oxygen to form superoxide [33]. The level of ROS production by macrophages directly determines the efficiency of phagocytosis. In the meantime, the development of chronic granulomatous disease is associated with defects in genes encoding components of NADPH oxidase, which determines the activity of the respiratory burst of phagocytes [34].

Antioxidant enzymes control cellular levels of ROS. Among them are superoxide dismutases (SOD), including copper-zinc SOD (Cu/Zn SOD, SOD1) [35]. The reduced abundance of functionally related proteins Ncf2 and Sod1 in PMJ2-R cells could be an indication of a weaker ability to implement the processes of respiratory burst by cells. In its turn, this might be associated with decreased phagocytosis intensity.

Engulfment and cell motility of protein 1 (Elmo1), which determines the efficiency of bacterial phagocytosis, was downregulated in PMJ2-R cells. This protein is present in the phagosome and enhances bacterial clearance due to differential regulation of lysosomal acidification and enzymatic activity. Macrophages depleted in Elmo1 demonstrate an increase in the pH of phagolysosomes and a decrease in cathepsin B activity, which leads to better bacterial survival [36]. Moreover, the Elmo1 protein works unidirectionally with RhoG and Rac1, other phagocytosis-associated proteins involved in the RhoG-Elmo1-Rac1 cascade [37].

In this cascade, small GTPase RhoG activates Rac1 via its effector ELMO and ELMO-binding protein Dock180, functioning as a Rac-specific guanine nucleotide exchange factor. One of the biological functions of this cascade is the regulation of cell migration, since small GTPases of the Rho family play an important role in this event by regulating many aspects of intracellular actin dynamics. In particular, Rac is a positive regulator of lamellipodia formation during migration. It should be noted that migration is an important characteristic of phagocytic cells [38].

At the same time, the expression of the Arhgap25 protein, which has the properties of Rac/Rho activating proteins (GAP), was comparable between PMJ2-R and PM (Figure 3). It has been shown that the Arhgap25 protein is specifically expressed in hematopoietic cells and serves as a significant negative regulator of the phagocytosis process, likely acting through the local modulation of the actin cytoskeleton [39,40].

In comparison with PM, the expression of the small G-protein Rac2 (Ras-related C3 botulinum toxin substrate 2) was reduced in PMJ2-R cells. Rac GTPases are thought to contribute to migration processes by transmitting signals from cell surface receptors to actin and microtubule cytoskeletons [41]. Reduced levels of F-actin, lack of podosomes (integrin-based adhesion sites), and a slightly reduced migration rate in comparison with wild-type macrophages have been described for Rac2 (−/−) macrophages [41]. Rac2 has been proposed as a positive regulator of integrin-dependent migration of macrophages [33].

Expression of the small G protein Cdc42 functionally associated with downregulated Elmo1 (Figure 3) was comparable between both cell lines. Like other small G-proteins, namely Rac1 and Rac2, the Cdc42 protein regulates a rearrangement of F-actin and membrane, which is necessary for macrophage phagocytosis mediated by the Fc-gamma receptor [42].

Apparently, Cdc42 activates the Wiskott–Aldrich syndrome protein (WASP) required for Fc-gamma receptor-mediated phagocytosis [43]. RNA silencing of Cdc42 expression, similar to an exposure of cells to the WASP inhibitor viscostatin, leads to significant changes in the phagocytic function of macrophages caused by defects in actin assembly and phagocytic cup formation. Moreover, the phenotypes of phagocytic cells, both with the decreased Cdc42 expression and exposed to viscostatin, are identical [44].

A comparable expression level of hematopoietic protein-1 (Hem-1, also known as Nck-associated protein 1-like (Nckap1l or Nap1l)) in PM and PMJ2-R cells was determined. This protein is also functionally associated with Elmo1 (Figure 3), the actin regulatory protein. Nckap1l is a part of the WAVE (WASP-family verprolin homologous protein) complex that transmits signals from activated Rac to stimulate F-actin polymerization in response to the immuno-receptor signaling [43]. Nckap1l is involved in the development, activation, proliferation, and homeostasis of lymphocytes, phagocytosis, and migration of neutrophils and macrophages [45,46]. Regulation of the F-actin polymerization in hematopoietic cells by Hem-1 has been shown, outlining its importance for biological processes in immune cells associated with the reorganization of the actin cytoskeleton [43]. One may conclude the same functional activity of Wasp-mediated processes in PMJ2-R cells and normal peritoneal macrophages.

Among other downregulated proteins associated with the process of phagocytosis in PMJ2-R cells, the Csk protein is of interest. Csk is a C-terminal Src-kinase, an enzyme phosphorylating tyrosine residue located at the C-terminus of Src family kinases. It suppresses the activity of these protein kinases; in particular, their pro-oncogenic activity decreases [47,48]. Thus, it is vital for the regulation of cell growth, differentiation, migration, and immune response. It serves as a negative regulator of the cell proliferation, transport of proteins through the plasma membranes of the Golgi complex. Apparently, a reduced Csk tyrosine kinase expression in PMJ2-R cells is a consequence of the immortalization of cells and is associated with their ability to proliferate actively. It is important to consider other functions of Src kinases, which may be altered in PMJ2-R due to decreased expression of Csk tyrosine protein kinase.

Additionally, the abundance of the receptor for activated protein kinase C1 (Rack1) was decreased in PMJ2-R cells. This protein serves as the anchor protein for protein kinase C (PKC). It can stabilize the active form of PKC and ensure its translocation to various sites within the cell. PKC is involved in many biological functions of monocytes, including phagocytosis. For example, inhibition of RACK1 by ZEBRA protein leads to PKC activity reduction, which, in turn, inhibits phagocytosis in phagocytes [49]. The inhibition of RACK1 activity by Yop proteins is associated with the ability of Yersinia pseudotuberculosis to resist phagocytosis. This phenomenon is called antiphagocytosis [50].

The ability of RACK1 to bind to a variety of signaling molecules, including members of the Src family, an integrin subunit, PDE45, and IGF-1 receptors, and to regulate the cell cycle, survival, adhesion, and migration has been shown [51]. This implies that RACK1 can function as a scaffold protein, mediating protein–protein interactions and facilitating tight regulation of cell functions, as well as cross-talks controlling various signaling cascades. In particular, a regulatory role of RACK1 has been described in VEGF-Flt1-dependent cell migration through the direct interaction with Flt1 (fms-like tyrosine kinase). In cells with reduced endogenous RACK1 expression attenuated by RNA, VEGF-driven migration was markedly suppressed, while proliferation was not affected and remained stable [52]. Thus, decreased amounts of the multifunctional proteins Csk and Rack1 might have an impact on a number of phagocytosis features in PMJ2-R cells.

The abundance of three other proteins associated with the process of phagocytosis, namely Anxa3, Lman2, and Pld4, were comparable in PM and PMJ2-R cells. Anxa3, or Annexin A3, is a protein of the annexin family whose members play roles in the regulation of cell growth and signal transduction pathways. Annexins bind phospholipids in a calcium-dependent manner. Although the exact function of annexins remains unknown, their involvement in vesicular transport and phagocytosis, including membrane–membrane or membrane–cytoskeleton interactions, is supported. Annexins are present on both the plasma membrane and phagosomes. Annexin A3 inhibits phospholipase A2 and the cleavage of inositol-1,2-cyclic phosphate to form inositol-1-phosphate. Annexin A3 has been found in early endosomes, and its amount remains the same at all times after formation [53,54].

The role of annexins in the modulation of apoptosis has been actively studied. Many annexin family proteins, including Anxa3, have been shown to inhibit apoptosis in tumor cells due to caspase-3 and caspase-8 repression (the result of Anxa3/JNK pathway activation) [55,56].

It has been shown that highly conserved annexins, including Anxa3, Anxa4, Anxa5, and Anxa13, can modulate phagocytosis of apoptotic cells due to their ability to bind to phosphatidylserine on their surface. Recognition of dying cells appears to be a common feature of most annexins. An individual repertoire of annexins associated with the cell surface of dying cells can perform an opsonin-like function, enhancing their recognition [57].

Lman2 (VIP36, 36 kDa vesicular integral membrane protein) is a transmembrane protein containing a lectin domain that serves as a cargo receptor for the Golgi to the endoplasmic reticulum transport and acts as a positive regulator of phagocytosis. Knockdown of endogenous VIP36 partially but reversibly inhibited phagocytosis, while overexpression of VIP36 led to a similarly reversible increase in phagocytosis in Raw 264.7 cells [58].

Pld4 (phospholipase D4) is a protein that has been found to be involved in the phagocytosis of activated microglia [59]. However, it was later discovered that it is also expressed by classically activated macrophages (macrophages M1). Moreover, the inhibition of Pld4 expression by siRNA leads to a significant decrease in pro-inflammatory cytokine secretion of M1 macrophages [60].

## 4. Materials and Methods

### 4.1. Cell Cultures

The murine macrophage cell line PMJ2-R (ATCC CRL2458TM) was cultivated in RPMI 1640 medium supplemented with 10% fetal bovine serum (FBS, both Biosera, Nuaille, France), 1% antibiotics-antimycotics (amphotericin B 0.25 mg/mL, penicillin G 100 units/mL, streptomycin 100 mg/mL), 1% L-alanyl-L-glutamine (all from Biowest, Nuaille, France), and 50 μM *beta*-mercaptoethanol (Sigma-Aldrich, Darmstadt, Germany). *Beta*-mercaptoethanol was not essential, but in vitro function of murine cells was found to benefit from its presence [61].

Primary peritoneal macrophages were derived from 2-month-old male mice C57BL/6. The animals were obtained from Velaz (Prague, Czech Republic) or the Breeding Center of the E. D. Goldberg Research Institute of Pharmacology and Regenerative Medicine (Tomsk, Russian Federation). The animals were kept in accordance with the rules of the European Convention for the Protection of Vertebrate Animals used for Experimental and Other Scientific Purposes (Strasburg, 1986). To isolate peritoneal macrophages, the animals were sacrificed, and peritoneal lavage was conducted with 10 mL cold PBS. The lavage fluid was centrifuged, and erythrocytes were lysed by the addition of 1 × RBC lysis buffer (eBioscences, Thermo Fisher Scientific, Eugene, OR, USA). The erythrocyte-free peritoneal cells were resuspended in RPMI 1640 medium supplemented with 10% heat-inactivated fetal bovine serum (FBS, both Biosera, Nuaille, France), L-alanyl-L-glutamine, 100 U/mL penicillin G, and 100 μg/mL streptomycin (all from Biowest, Nuaille, France), 50 μM beta-mercaptoethanol (Sigma-Aldrich, Darmstadt, Germany) seeded into T25 cell culture flasks, and incubated at 37 °C and 5% CO_2_ for 4 h. After 4 h incubation, non-adherent cells were removed by repeated flushing of the flasks with culture medium to obtain pure peritoneal macrophages. For proteomic analyses, cells were washed with PBS. For phagocytosis analysis, cells were centrifuged and the cell pellet was resuspended in 1 mL warm RPMI 1640 medium supplemented with 10% FBS, 1% antibiotics-antimycotics (amphotericin B 0.25 mg/mL, penicillin G 100 units/mL, streptomycin 100 mg/mL), 1% L-alanyl-L-glutamine.

### 4.2. Protein Extraction and Sample Preparation for MS Analysis

Cells (about 1 × 10^6^) were washed thoroughly in PBS. Cell lysis was performed in 1 mL of 2% SDS and 100 mM DTT in 0.1 M Tris-HCl (pH 7.6) at room temperature with a brief sonication to reduce the viscosity of the lysate. Total protein content in samples was measured according to the BCA method [62]. A total protein amount of 100 μg for each sample was used for tryptic digestion according to the common FASP protocol [63]. Briefly, detergents in the samples were exchanged with 100 mM Tris-HCl (pH 8.5) using Microcon filters (10 kDa cut off, Millipore, Bedford, MA, USA).

Protein disulfide bridges were reduced with 100 mM DTT in 100 mM Tris-HCl (pH 8.5), alkylation of thiols was performed with 55 mM iodoacetamide in 8 M urea/100 mM Tris-HCl (pH 8.5). Digestion with trypsin (Sequencing Grade Modified, Promega, Madison, WI, USA) to protein ratio of 1:100 was carried out overnight at 37 °C in a 50 mM tetraethyl ammonium bicarbonate (pH 8.5). To obtain the peptide solution, the filtered samples were centrifuged at 11,000× *g* for 15 min in a thermostated centrifuge at 20 °C. The filters were then washed with 50 mL of 30% formic acid solution by centrifugation at 11,000× *g* for 15 min in a thermostat centrifuge at 20 °C. The filtrates were dried in a vacuum concentrator and dissolved in 20 mL of 5% formic acid for subsequent MS analysis.

One microgram of peptides in a volume of 1 µl was loaded directly onto the 15-cm long C18 column (Acclaim^®^ PepMap™ RSLC inner diameter of 75 μm, Thermo Fisher Scientific, Rockwell, IL, USA) at a flow rate of 0.3 µL/min for 12 min in an isocratic mode of Mobile Phase C (2% acetonitrile, 0.1% formic acid). Then, the peptides were separated with high-performance liquid chromatography (HPLC, Ultimate 3000 Nano LC System, Thermo Scientific, Germering, Germany) in a 15 cm long C18 column (Acclaim^®^ PepMap™ RSLC inner diameter of 75 μm, Thermo Fisher Scientific, Rockwell, IL, USA). The peptides were eluted with a gradient of buffer B (80% acetonitrile, 0.1% formic acid) at a flow rate of 0.3 μL/min. Total run time was 130 min and included 12 min of column equilibration to buffer A (0.1% formic acid), gradient from 5 to 35% of buffer B over 95 min, 6 min to reach 99% of buffer B, flushing 10 min with 99% of buffer B, and 7 min re-equilibration to buffer A.

MS analysis was performed at least in triplicate with a Q Exactive HF-X mass spectrometer (Q Exactive HF-X Hybrid Quadrupole-OrbitrapTM Mass spectrometer, Thermo Fisher Scientific, Bremen, Germany). The temperature of capillary was 240 °C and the voltage at the emitter was 2.1 kV. Mass spectra were acquired at a resolution of 120,000 (MS) in a range of 300–1500 m/z. Tandem mass spectra of fragments were acquired at a resolution of 15,000 (MS/MS) in the range from 140 m/z to m/z value determined by a charge state of the precursor, but no more than 2000 m/z. The maximum integration time was 50 ms and 110 ms for precursor and fragment ions, correspondently. AGC target for precursor and fragment ions were set to 1 × 10^6^ and 2 × 10^5^, correspondently. An isolation intensity threshold of 50,000 counts was determined for precursor’s selection and up to top 20 precursors were chosen for fragmentation with high-energy collisional dissociation (HCD) at 29 NCE. Precursors with a charge state of +1 and more than +5 were rejected and all measured precursors were dynamically excluded from triggering of a subsequent MS/MS for 20 s.

Mass spectrometric measurements were performed using the equipment of “Human Proteome” Core Facility of the Institute of Biomedical Chemistry (Moscow, Russia).

The MS/MS spectra in a RAW format were processed in SearchGUI v.3.3.20 [64]. Obtained data were deposited to the ProteomeXchange Consortium via the PRIDE [65] partner repository with the dataset identifier PXD022133.

### 4.3. Protein Identification

Protein identification was conducted against a concatenated target/decoy version of the mouse complement of the UniProtKB. The decoy sequences were created by reversing the target sequences in SearchGUI v.3.3.20. The identification settings were as follows: trypsin specific with a maximum of 2 missed cleavages; 5.0 ppm as MS1 and 0.01 Da as MS2 tolerances; fixed modification: carbamidomethylation (Cys); variable modifications: N-terminal proteins acetylation, and methionine oxidation (Met). Peptide Spectrum Matches (PSMs), peptides, and proteins were validated at a 1.0% false discovery rate estimated using the decoy hit distribution.

### 4.4. Data Analysis

Progenesis-QI LC-MS software (version 4.1, Nonlinear Dynamics, Newcastle upon Tyne, UK) was used for a label-free quantification.

The raw files from the mass spectrometer were uploaded onto the ProgenesisQI for proteomics software package. The LC-MS data were aligned according to the manufacturer’s recommendations.

Proteins for the analysis were selected if number of identified peptides inside each biological replicate was greater than two. Qualitative diversity of proteins among samples was depicted using the UpSetR [66]. The Wilcoxon test was applied to compare the median of the emPAI values from three biological replicates of series PMJ2-R to the median of emPAI values from three biological replicates of series PMs independently [67]. The thresholds for significantly changed proteins were set to *p* value < 0.01 and |log2FC| > 2. Thus, upregulated proteins had *p* < 0.01 and log2FC > 2, and downregulated proteins had *p* < 0.01 and log2FC < −2. The proteins were considered as “unchanged” with applied filter by the fold changes without taking into account statistical significance: 0.5 < fold change < 2.

Functional analysis of up- and downregulated proteins was performed using “Mapping to ontology” workflow of the GeneXplain platform (https://genexplain.com/genexplain-platform/, accessed on 22 May 2021). A functional classification module was used for the annotation of differentially expressed genes in terms of Gene Ontology. The algorithm of the functional analysis is intended to detect statistically significant representation of certain functional groups of genes or proteins among all genes/proteins of input sets. Statistical significance of the classification was estimated with an adjusted *p* value, and only statistically significant GO groups with adjusted *p* values < 0.001were considered.

Protein networks were built using cytoscape StringApp [68] and ClusterMaker [69].

### 4.5. Phagocytosis Assay

C57BL/6 peritoneal macrophages and PMJ2-R cells were cultured in the DMEM/F12 medium supplemented with 10% of FBS and 1% of penicillin/streptomycin in 250 mL culture flasks at 37 °C in humidified air with 5% CO_2_. Cells were seeded onto washed sterilized coverslips at the density of 4 × 10^4^ per 1 cm^2^ the night prior to the experiment. For stimulation of phagocytosis, PMJ2-R cells were incubated with 20 ng/mL IFN-gamma for 24 h before the experiment.

Then, 40-kDa FITC-labeled dextran or FITC-labeled opsonized zymosan were dissolved directly in a fresh complete culture medium to obtain working solutions containing dextran at a concentration of 100 μg/mL or zymosan at 1 mg/mL. Cells were incubated in FITC-dextran or FITC-zymosan solution for 2 h at 37 °C in an atmosphere of 5% CO_2_. Negative control cells were incubated in FITC-dextran or FITC-zymosan solution at 4 °C (for live cells endocytosis and phagocytosis block) [70]. All cells were gently washed 3 times with PBS. Coverslips were mounted on slides with Prolong medium (Invitrogen by Thermo Fisher Scientific, Eugene, OR, USA), and images were immediately captured with a ZOE™ Fluorescent Cell Imager (Bio-Rad, Hercules, CA, USA).

The obtained images were processed in CellProfiler (Broad Institute of Harvard and MIT; MA, USA) [71] (see Supplementary File 2). At least 150 cells were analyzed for each sample. Relative fluorescence intensity of FITC-dextran per cell, a portion of phagocytic cells, and zymosan granules per phagocytic cell were counted. Differences between the groups were tested using Student’s *t*-test with the Benjamini–Hochberg procedure for multiple comparisons. Differences were considered significant at *p* < 0.05.

## 5. Conclusions

The study of the PMJ2-R cells’ proteome provided evidence of significant differences in the protein profiles of immortalized cells and normal peritoneal mouse macrophages. Analysis of quantitative proteomic data revealed significant downregulation of proteins involved in protein synthesis, cellular metabolism, the implementation of redox reactions, and the cell’s response to oxidative stress and hypoxia.

Proteins involved in important processes such as respiratory burst, reorganization of the cytoskeleton associated with the processes of cell migration, clathrin exchange and phagocytosis (absorption) itself, IgG-mediated phagocytosis, as well as a number of proteins that regulate the activity of phagocytosis (Elmo1, Gsn, Hspa8, Itgb1, Ncf2, Rac2, Rack1, Sirpa, Sod1, C3, Msr1), were reduced in the PMJ2-R cells. Changes in the protein profile of PMJ2-R cells resulted in their reduced phagocytic ability compared to PM (zymosan granules), which could be slightly restored by cells exposure to IFN-gamma. This study provides valuable information for researchers in the field of macrophage physiology. Obtained data should be taken into account by researchers when choosing the optimal model of macrophage-like cell line.

## Figures and Tables

**Table 1:** Top 15 up- and downregulated proteins in PMJ2-R cells compared to PM.

Accession in UniProtKB	Protein Name	Gene Name	*p*-Value *	Fold Change	Function/Effect **
***Upregulated proteins:***					
Q9QYX7	Protein piccolo	*Pclo*	2.52 × 10^−7^	3727.85	Scaffold protein of the presynaptic cytomatrix at the active zone. Participates in the formation of Golgi-derived membranous organelles termed Piccolo-Bassoon transport vesicles. Mediates a synapse communication to the nucleus, leading to a reconfiguration of gene expression by association with the transcriptional corepressor CTBP1 and by subsequent reduction in its pool available for a nuclear import.
P30204	Macrophage scavenger receptor types I and II	*Msr1*	2.28 × 10^−6^	839.75	Involved in phagocytosis, engulfment.
Q2XU92	Long-chain-fatty-acid-CoA ligase ACSBG2	*Acsbg2*	4.01 × 10^−7^	314.92	Involved in cell differentiation, fatty acid metabolic process.
Q8CGN8	Small proline-rich protein 4	*Sprr4*	4.36 × 10^−6^	303.20	Involved in keratinization.
Q91WM1	Spermatid perinuclear RNA-binding protein	*Strbp*	1.17 × 10^−3^	262.87	Plays a role in the cell growth regulation (by similarity). Binds to double-stranded DNA and RNA.
Q9WV27	Sodium/potassium-transporting ATPase subunit alpha-4	*Atp1a4*	2.07 × 10^−8^	191.24	Catalytic component of the active enzyme, which catalyzes the hydrolysis of ATP coupled with the exchange of sodium and potassium ions across the plasma membrane. Responsible for the creation of the electrochemical gradient of sodium and potassium ions, providing the energy for active transport of various nutrients.
Q62383	Transcription elongation factor SPT6	*Supt6*	4.50 × 10^−5^	190.45	Binds to histone H3 and plays a key role in the regulation of transcription elongation and mRNA processing.
Q6GQT1	Alpha-2-macroglobulin-P	*A2m*	1.64 × 10^−5^	179.54	Serine protease inhibitor, involved in tumor necrosis factor binding.
A2AQ07	Tubulin beta-1 chain	*Tubb1*	1.73 × 10^−6^	112.87	Structural constituent of cytoskeleton, mitotic cell cycle.
Q6ZQ06	Centrosomal protein of 162 kDa	*Cep162*	3.77 × 10^−15^	95.63	Required to promote assembly of the transition zone in primary cilia. Acts by specifically recognizing and binding the axonemal microtubule.
Q6IFX2	Keratin, type I cytoskeletal 42	*Krt42*	8.83 × 10^−8^	95.14	Part of intermediate filament, structural molecule activity.
Q8BI79	Coiled-coil domain-containing protein 40	*Ccdc40*	6.66 × 10^−16^	73.11	Plays a central role in motility in cilia and flagella.
Q99LB6	Methionine adenosyltransferase 2 subunit beta	*Mat2b*	2.24 × 10^−7^	71.02	Methionine adenosyltransferase regulator activity, interacts with diverse chromatin regulators and methyltransferases, serves as a transcriptional corepressor of Maf oncoprotein.
Q7TMM9	Tubulin beta-2A chain	*Tubb2a*	7.42 × 10^−5^	66.53	Microtubule cytoskeleton organization, structural constituent of cytoskeleton, involved in mitotic cell cycle.
P01027	Complement C3	*C3*	4.00 × 10^−12^	61.62	Involved in a positive regulation of phagocytosis and apoptotic cell clearance.
***Downregulated proteins:***					
Q9D903	Probable rRNA-processing protein EBP2	*Ebna1bp2*	6.93 × 10^−8^	−1562.14	Involved in ribosomal large subunit biogenesis, rRNA processing.
Q80VJ3	2′-deoxynucleoside 5′-phosphate N-hydrolase 1	*Dnph1*	3.45 × 10^−7^	−436.33	Involved in a nucleoside metabolic process, cell differentiation, positive regulation of cell growth.
Q7TNC4	Putative RNA-binding protein Luc7-like 2	*Luc7l2*	1.81 × 10^−5^	−385.67	Involved in enzyme binding, mRNA binding, mRNA splice site selection.
O70318	Band 4.1-like protein 2	*Epb41l2*	6.03 × 10^−9^	−301.68	Involved in actin cytoskeleton organization, cell cycle, cell division.
Q91VM5	RNA binding motif protein, X-linked-like-1	*Rbmxl1*	5.82 ×10^−8^	−297.73	Involved in mRNA processing, mRNA splicing, mRNA splice site selection.
P13541	Myosin-3	*Myh3*	7.65 × 10^−6^	−229.84	Enables actin filament binding, involved in skeletal muscle contraction, ATP binding.
Q8K1J6	CCA tRNA nucleotidyltransferase 1, mitochondrial	*Trnt1*	1.39 × 10^−6^	−195.84	Adds and repairs the conserved 3′-CCA sequence necessary for the attachment of amino acids to the 3′ terminus of tRNA molecules, involved in tRNA processing.
Q91YP2	Neurolysin, mitochondrial	*Nln*	1.95 × 10^−7^	−186.82	Hydrolyzes oligopeptides, involved in a regulation of gluconeogenesis.
Q8C052	Microtubule-associated protein 1S	*Map1s*	9.31 × 10^−7^	−162.55	Mediates aggregation of mitochondria resulting in the cell death and genomic destruction.
O70591	Prefoldin subunit 2	*Pfdn2*	7.68 × 10^−5^	−124.24	Involved in protein folding, positive regulation of cytoskeleton organization.
P28658	Ataxin-10	*Atxn10*	1.38 × 10^−5^	−123.10	May play a role in the maintenance of a critical intracellular glycosylation level and homeostasis.
P62983	Ubiquitin-40S ribosomal protein S27a	*Rps27a*	7.02 × 10^−4^	−81.40	Involved in protein ubiquitination, translation.
E9PYG6	Protein Rasa1	*Rasa1*	4.98 ×10^−5^	−77.80	Involved in regulation of GTPase activity, negative regulation of apoptotic process.
Q99P72	Reticulon-4	*Rtn4*	7.85 × 10^−7^	−71.74	Required to induce the formation and stabilization of endoplasmic reticulum tubules.
Q8JZM0	Dimethyladenosine transferase 1, mitochondrial	*Tfb1m*	8.12 ×10^−3^	−71.35	Specifically dimethylates mitochondrial 12S rRNA at the conserved stem loop, required for basal transcription of mitochondrial DNA, stimulates transcription independently of the methyltransferase activity.

* *p*-value—significance level for one-way ANOVA; ** Function/effect was derived from UniProtKB protein description.

**Table 2:** Phagocytic activity of PMJ2-R and PM cells.

Cell Line	PM	PMJ2-R	PMJ2-R+IFNyγ
Percentage of phagocytic cells, %	79.6 ± 6.7	5.1 ± 2.4 *	21.5 ± 7.7 *^,#^
Zymosan granules per phagocytic cell	9.2 ± 3.2	1.7 ± 0.7 *	4.1 ± 1.1 *^,#^
RFI of FITC-dextran per cell	1.00 ± 0.19	0.27 ± 0.15 *	0.32 ± 0.17 *

* Differences are significant relative to the PM group (*p* < 0.05). ^#^ Differences are significant relative to the PMJ2-R group (*p* < 0.05). RFI—relative fluorescence intensity.

## Data Availability

Obtained data were deposited to the ProteomeXchange Consortium via the PRIDE partner repository with the dataset identifier PXD022133.

## Supplementary Materials

The following are available online at https://www.mdpi.com/article/10.3390/ijms22126323/s1.
