# Supplementary material for: Proteome Profiling of PMJ2-R and Primary Peritoneal Macrophages
Source: Int J Mol Sci. 2021 Jun 12;22(12):6323. doi: 10.3390/ijms22126323 (PMC8231560; doi:10.3390/ijms22126323)
Supplement: Supplementary file 1 [file ijms-22-06323-s001.zip › Supplementary2.pdf]

CellProfiler protocol to count FITC-Zymosan phagocytosis was as follows:
1. Split Color image to separate gray channels for nuclei and zymosan (ColorToGray)
2. Identify all nuclei objects in grayscale images (IdentifyPrimaryObjects)
3. Identify all zymosan objects in grayscale images (IdentifyPrimaryObjects)
4. Relate zymosan objects to nuclei objects (RelateObjects)
5. Export measurements to spreadsheet (ExportToSpreadsheet)
6. Calculate nuclei with children zymosan count and mean zymosan particles per cell.

CellProfiler protocol to count FITC-dextran endocytosis was as follows:
1. Split Color image to separate gray channels for nuclei and dextran (ColorToGray)
2. Identify all nuclei objects in grayscale image (IdentifyPrimaryObjects)
3. Identify FITC-dextran regions in grayscale images around nuclei (IdentifySecondaryObjects)
4. Measure fluorescence intensity of FITC-dextran per cell (SecondaryObjects) (MeasureObjectIntensity)
5. Export measurements to spreadsheet (ExportToSpreadsheet)
6. Calculate mean fluorescence intensity per cell and normalize to control group.
